# Testing for physical validity in molecular simulations

**DOI:** 10.1371/journal.pone.0202764

**Published:** 2018-09-06

**Authors:** Pascal T. Merz, Michael R. Shirts

**Affiliations:** Department of Chemical and Biological Engineering, University of Colorado Boulder, Boulder, CO 80309, United States of America; Hong Kong University of Science and Technology, HONG KONG

## Abstract

Advances in recent years have made molecular dynamics (MD) and Monte Carlo (MC) simulations powerful tools in molecular-level research, allowing the prediction of experimental observables in the study of systems such as proteins, membranes, and polymeric materials. However, the quality of any prediction based on molecular dynamics results will strongly depend on the validity of underlying physical assumptions. Unphysical behavior of simulations can have significant influence on the results and reproducibility of these simulations, such as folding of proteins and DNA or properties of lipid bilayers determined by cutoff treatment, dynamics of peptides and polymers affected by the choice of thermostat, or liquid properties depending on the simulation time step. Motivated by such examples, we propose a two-fold approach to increase the robustness of molecular simulations. The first part of this approach involves tests which can be performed by the users of MD programs on their respective systems and setups. We present a number of tests of different complexity, ranging from simple post-processing analysis to more involved tests requiring additional simulations. These tests are shown to significantly increase the reliability of MD simulations by catching a number of common simulation errors violating physical assumptions, such as non-conservative integrators, deviations from the Boltzmann ensemble, and lack of ergodicity between degrees of freedom. To make the usage as easy as possible, we have developed an open-source and platform-independent Python library (https://physical-validation.readthedocs.io) implementing these tests. The second part of the approach involves testing for code correctness. While unphysical behavior can be due to poor or incompatible choices of parameters by the user, it can just as well originate in coding errors within the program. We therefore propose to include physical validation tests in the code-checking mechanism of MD software packages. We have implemented such a validation for the GROMACS software package, ensuring that every major release passes a number of physical sanity checks performed on selected representative systems before shipping. It is, to our knowledge, the first major molecular mechanics software package to run such validation routinely. The tests are, as the rest of the package, open source software, and can be adapted for other software packages.

## Introduction

Advances in recent years have made molecular dynamics (MD) simulations a powerful tool in molecular-level research, allowing the prediction of experimental observables in the study of systems such as proteins, drug targets [1, 2], membranes and polymers [3]. However, the quality of any prediction based on molecular dynamics results will strongly depend on the validity of underlying physical assumptions [4].

There are two main sources of errors that violate physical assumptions, the programmer and the user. Errors introduced by the programmer can range from simple bugs to hard-to-catch corner cases when combining seldom used techniques or running the program on exotic hardware. A perfect implementation alone does, however, not guarantee that the program is applied correctly. Using a method or a combination of methods inappropriate for the investigated system will lead to wrong results even in the hypothetical case of a perfect program. Two trends are increasing the risk for such unsuitable use. First, modern molecular simulation program packages allow for a multitude of methods to be applied, and are increasingly allowing users to interact with low-level functionality via APIs, further increasing the risk for inappropriate applications. Additionally, the advances in both hardware and software over the past years have made MD simulations available to many users to examine complicated problems, many of whom are not experts in molecular simulations.

The lack of physical validity can strongly influence the results obtained from molecular simulations in both biomolecular and materials applications, and lead to a significant lack of reproducibility in the field. For example, Ni and Baumketner found that the treatment of non-bonded interaction cutoffs affects the folding of biomolecules such as proteins and DNA fragments in reaction-field simulations [5]. The truncation of electrostatic interactions can meaningfully influence the properties of lipid bilayers such as enhanced ordering and decreased area per lipid [6, 7]. Wong-ekkabut *et al*. noted that reported water flow through nanotubes could be attributed to the use of charge-group cutoff and the lack of buffers in pairlist generation [8]. They further noted an influence of the thermostatting algorithm on the flux. Similar artifacts causing continuous water flow were found in MD simulations of amyloid crystals [9]. By comparing results from different MD packages (but identical cutoff scheme), Bonthuis *et al*. found that spurious flow effects of water in static electric fields due to force-cutoff were implementation-related [10]. The treatment of long-range electrostatic forces was also shown to influence the free energy of transfer of tryptophan analogs [11] and the energetic, structural and dielectric properties of water [12]. The choice of thermostat does not only determine the kinetic energy distribution sampled [13], but was also shown to influence the distribution of temperature between degrees of freedom [14, 15] (“flying ice cube effect”). The impact of this effect on physical observables was demonstrated by Leyssale and Vignoles [15] finding graphitization of nanodiamonds severely impaired by the loss of internal kinetic energy. The coupling of solute and solvent to separate heat baths has been used to mitigate the hot-solvent/cold-solute problem [16]. It was later shown, however, that this approach can significantly impact the dynamics of macromolecules [17, 18], affecting for example the flip rates of peptides [17]. Winger *et al*. reported that the choice of too large timesteps for systems of pure coarse-grained water and hexadecane does impact properties such as the density, the potential energy, or the excess free energy [19]. These are just as small sampling of the many studies that show how incorrect simulation choices can significantly affect the physical validity of the simulations.

A two-fold approach on testing for physical validity can strongly increase the robustness, reliability, and reproducibility of molecular simulations. The first part of this approach involves tests which can be easily performed by the users of MD programs on their respective systems and setups. We present a number of tests of different complexity, ranging from simple post-processing analysis to more involved tests requiring additional simulations. In this work, we show that these tests can significantly increase the reliability of MD simulations by catching a number of common simulation errors violating physical assumptions, such as non-conservative integrators, deviations from the Boltzmann ensemble, and lack of ergodicity between degrees of freedom. To make them as easy as possible to use, we developed an open-source and platform-independent Python library containing these tests (see https://physical-validation.readthedocs.io).

The second part of this approach involves testing for correctness of the simulation packages themselves. While unphysical behavior can be due to poor or incompatible choices of parameters by the user, it can just as well originate in coding or algorithm errors within the program. Traditional software testing focuses on the correctness of code, but it cannot generally guarantee physical correctness. Violation of physical assumptions can occur in perfectly valid code, e.g. by operating outside the range of validity of a model, or by allowing the combination of incompatible methods or parameters. We therefore propose to include physical validation tests in the code-checking mechanism of MD software packages. We have implemented such a validation for the GROMACS software package [20, 21] ensuring that every major releases passes a number of physical sanity checks performed on selected representative systems before shipping. It is, to our knowledge, the first major molecular mechanics software package available for general usage to run such validation routinely. (We have, however, learned through personal communication with Bill Swope of IBM Research that ‘Blue Matter’, the internal molecular dynamics code that IBM Research used on the Blue Waters petascale computing project, did use an automated physical validation suite with some overlap with the current suite, but neither the suite nor the code was ever made publicly available.) The tests are, as the rest of the package, open source software, and can be adapted for other software packages.

## Validation of physical assumptions

### Integrator validation

Integrators routinely used in MD simulations do not sample the actual Hamiltonian H of the system, but a closely related *shadow Hamiltonian* [22] $\tilde{H}$. For symplectic second-order integration algorithms such as velocity-verlet [23] or leap-frog [24] using a timestep Δ*t*, the physical and the shadow Hamiltonian are related by

$$\begin{array}{c} \tilde{H}=H+O\;(\Delta t^{2})\;. \end{array}$$ (1)

The shadow Hamiltonian is generally not known analytically. Its expectation value is constant,

$$\begin{array}{c} \left\langle \tilde{H}\right\rangle =\tilde{E}\;, \end{array}$$ (2)

where the shadow energy, $\tilde{E}$, is a constant which differs from the physical constant energy 〈H〉=E by a small bias factor that depends on the details of the simulation, notably on the chosen time step, but not on the length of the simulation. The expectation value of the physical Hamiltonian (“the total energy”) of a constant energy simulation using a symplectic second-order integration algorithm will hence be

$$\begin{array}{c} {\left\langle H\right\rangle }_{\Delta t}=\tilde{E}\;, \end{array}$$ (3)

while its instantaneous value will deviate from the average on the order of Δ*t*^2^,

$$\begin{array}{c} H=\tilde{E}+O\;(\Delta t^{2})\;. \end{array}$$ (4)

The fluctuation in Δ*t*^2^ of the Hamiltonian with respect to the average value can be used to asses simulation correctness. Combining Eqs 3 and 4, we see that the fluctuations of the instantaneous H around its average value 〈H〉 must be of the order of Δ*t*^2^,

$$\begin{array}{c} \sigma (H)∼\Delta t^{2}\;. \end{array}$$ (5)

Comparing the fluctuations around the average total energy of two (otherwise identical) simulation runs performed at different timesteps, one therefore expects their ratio to be dependent on the ratio of their squared timesteps,

$$\begin{array}{c} \frac{\sigma (H(\Delta t_{1}))}{\sigma (H(\Delta t_{2}))}=\frac{\Delta t_{1}^{2}}{\Delta t_{2}^{2}}\;. \end{array}$$ (6)

A deviation from the expected fluctuation ratio in Eq 6 hints at some inaccuracy in the simulation protocol. Effectively, it means that the simulation is not sampling the expected shadow Hamiltonian. There are, however, many possible causes for such inaccuracies, such as discontinuities in the potential or the forces, imprecisions in constraints, or a wrong integration algorithm. While the failure of such a test cannot necessarily determine the actual source of error, they permit to detect problems in the simulation protocol, which can be introduced by coding bugs or inappropriate simulation parameters.

### Kinetic energy validation

The distribution of the kinetic energy is well-defined in any constant temperature ensemble. The three components *p*_*i*,*x*_ of the momentum vector momentum ***p***_*i*_ of any particle *i* with mass *m*_*i*_ of a system in equilibrium at constant temperature *T* are individually normally distributed [25] with mean zero and variance *m*_*i*_*k*_*B*_*T*, having a probability density function (pdf) given by

$$\begin{array}{c} f\left(p_{i,x}\right)=\sqrt{\frac{1}{2\pi m_{i}k_{B}T}}e^{-\frac{p_{i,x}^{2}}{2m_{i}k_{B}T}}\;. \end{array}$$ (7)

The kinetic energy of a system of particles is the weighted sum of their individual momenta,

$$K\left(p\right)=\frac{1}{2}\sum_{i}\frac{1}{m_{i}}p_{i}\cdot p_{i}=\frac{1}{2}\sum_{i,x}{\left(\frac{p_{i,x}}{\sqrt{m_{i}}}\right)}^{2}.$$ (8)

The kinetic energy is hence the sum of squared variables individually normal-distributed with variance *k*_*B*_*T*. Sums of independent normally-distributed variables with zero mean are well-known to necessarily follow a gamma distribution, with parameters *α* (the *shape*) and *θ* (the *scale*) and pdf given by

$$\begin{array}{c} f_{\gamma }\left(x|\alpha ,\theta \right)=\frac{\theta^{-\alpha }}{\Gamma (\alpha )}x^{\alpha -1}e^{-\frac{x}{\theta }}\;. \end{array}$$ (9)

For *N* independent variables with variance *k*_*B*_*T*, the parameters take the values of *α* = *N*/2 and *θ* = *k*_*B*_*T*. This leads to the probability density function (pdf) of the kinetic energy,

$$\begin{array}{c} f\left(K|N,T\right)=\frac{\beta^{N/2}}{\Gamma (\frac{N}{2})}K^{N/2-1}e^{-\beta K}\;, \end{array}$$ (10)

where *N* denotes the number of degrees of freedom of the system, and *β* = (*k*_*B*_*T*)^−1^.

#### Kinetic energy separation

Not only the total kinetic energy of a system, but also the kinetic energy of any of its subset of degrees of freedom follows the distribution described in Eq 10, with the *α* value corresponding to the subset’s number of degrees of freedom divided by two. This follows from the fact that the kinetic energies of orthogonal degrees of freedom are uncorrelated and that the sum in Eq 8 can go over any subset of degrees of freedoms in the system, a principle known as *equipartition*. Separation of the kinetic energy allows to validate that functional parts of the system, such as solute and solvent or different components of liquid mixtures, have the proper kinetic energy distribution. Separations can also be applied spatially or randomly to arbitrary systems as a general sanity check. Any kinetic energy test that is proposed can (and should!) hence also be applied to subsets of a system.

An especially useful application of the equipartition check separates the molecular degrees of freedom into their translational, rotational, and internal components. Temperature control algorithms scaling the velocities of decoupled degrees of freedom uniformly were found to systematically move energy from fast to slow degrees of freedom [14] (most famously observed as the “flying ice cube” effect). Such systems can have correct distributions of total kinetic energy, which is, however, only due to compensation effects between too hot (typically translational or rotational) and too cold (typically internal) degrees of freedom. Condensed phase systems are generally assumed to have enough energy exchange between degrees of freedom to mitigate the effect of the velocity scaling. This is, however, an assumption worth checking in general, and especially for systems with low density. Details on the separation of molecular degrees of freedom are given in Ref. [26] and in S2 Text.

The tests presented here can be implemented independent of the exact estimator used for the kinetic energy. Eastwood *et al*. [27] have shown that kinetic energy estimators exclusively based on either the integrated momenta or the time-derivative of the positions can lead to violations of the equipartition principle when using integrators with finite timestep size. Unfortunately, the more robust estimator proposed by Eastwood *et al*. is rarely available in standard simulation packages. The discussion and implementation of alternative kinetic energy estimators within software packages is not in the scope of this work, however. Since the discussed tests are derived from physical principles, they will be valid up to the numerical error in the kinetic energy estimate.

#### Full distribution test

The Kolmogorov-Smirnov (K-S) test [28, 29] makes it possible to check simulated kinetic energy distributions versus the expected distribution. It has robust implementations available, *e.g*. in the SciPy library [30]. The K-S test is a nonparametric test assessing the equality of distributions. In this work, it is used to compare the observed distribution of the sample with the reference distribution described in Eq 10. It operates under the null hypothesis that the presented data set was drawn from the reference distribution. The test involves constructing a Kolmogorov distribution by computing the distance between the cumulative distribution functions of the distributions, and calculating the *p*-value from the critical values of the Kolmogorov distribution. This *p*-value can be identified as the probability to observe a sample at least as extreme as the one at hand under the null hypothesis. As an example, a *p*-value of 0.02 means that the probability to observe a sample at least as extreme when drawing a new sample of the same size from the reference distribution is 2%.

The K-S test does have some disadvantages, which we judge not to be of significance for the current application. Specifically, the K-S test is not valid when testing distributions in which the parameters of the distribution are inferred from the data set [31], and having declining sensitivity around the tails of the distribution. Within the scope of testing the kinetic energy of molecular simulations, we judge the advantages of a robust and readily available implementation to be important, and the disadvantages mentioned above to be largely insignificant. The parameters (the number of degrees of freedom and the target temperature) are never inferred from the trajectory of kinetic energies in our implementation, but rather from the actual system and the chosen target temperature of the coupling algorithm. Very high tail sensitivity is not generally desirable for most simulations of moderate length, as statistical noise in the tails of the empirical distribution might be flagged by the rest of the test even when the simulations is well-behaved. However, if particular simulations were calculating properties especially sensitive to kinetic-energy tail behavior, choosing a different statistical test may be advisable.

**Sample-size, system-size and temperature dependence of the statistical kinetic energy test** The K-S test can be too sensitive for certain applications. While the underlying theory defines an unambiguous distribution, simulations introduce numerical artifacts which can result in a slightly modified distribution. Very accurate tests are desirable when testing code under well-controlled conditions, but might yield false positives in less sensitive applications. It might for example not always be desirable to flag deviations from the desired kinetic energy distribution which are smaller in magnitude than other well-controlled approximations such as the interaction cutoff or the treatment of bond constraints.

The sensitivity of statistical tests like the K-S test increases with the number of samples [32]. With only a few observations, it is not possible to conclusively discriminate between closely related distributions. However, as the size of a sample approaches infinity, the test will be able to detect even slightest deviations in the parameters of the generating distribution versus the tested distribution.

**Fig 1:**
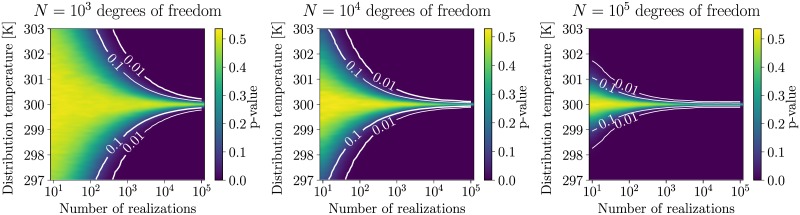
Sensitivity of the K-S test. The sensitivity of the K-S test depends on the number of degrees of freedom and the number of realizations. Results from validating 25 different sample sizes (x-axis) drawn from a gamma distributions at 101 different temperatures (y-axis) but fixed number of degrees of freedom (10^3^, 10^4^, and 10^5^, for the first, the second and the third panel, respectively), against a gamma distribution at 300 K and the same number of degrees of freedom. All tests were repeated 1000 times. The color bars indicate the average *p*-value obtained from the tests, with two contour lines indicating the *p* = 10% and *p* = 1% levels. The values between the sampled data points were approximated using bi-cubic interpolation.

The sensitivity of the K-S test of the kinetic energy distribution also depends on the number of degrees of freedom and the target temperature. Fig 1 illustrates this by drawing a number of samples (x-axis) from a gamma distribution at a specific temperature (y-axis), and testing it against an analytical gamma distribution at a specific temperature (300 K). The number of degrees of freedom is thereby the same for the generating distribution and the reference distribution, namely 10^3^, 10^4^, and 10^5^ for the first, the second and the third panel, respectively.

Larger sample size, lower temperature, or more degrees of freedom all make the test more sensitive. A deviation of 0.5 K in average temperature might well be within a typical cutoff of *p* ≥ 0.05 when analyzing 1000 samples, but might yield a *p* value very close to zero when increasing sample size by a factor 10. Small deviations in the sampled ensemble can have many reasons, including incorrect temperature-control algorithms, but also numerical noise from interaction cutoff schemes and long-range corrections, integration timesteps, or constraining algorithms. When developing or implementing new temperature control algorithms in a controlled test environment keeping errors from other sources negligible, a very high sensibility is certainly desirable. In many other, real-world applications, however, a deviation insignificant in comparison with other sources of inaccuracies might be enough to flag long simulation trajectories of large systems as not gamma distributed. It is therefore important to compare the pure numerical result obtained from the statistical distribution test to other indicators, such as the mean and the variance or even a visual inspection of the observed distribution compared to the expected distribution, and choose the correct indicator for the application at hand.

#### Validation of mean and standard deviation

The mean and the standard deviation of the simulated kinetic energy trajectory can be used to devise a less sensitive but more robust and intuitive alternative to the K-S test. The gamma distribution of the kinetic energy has closed formulas for the mean *μ* and the standard deviation *σ*,

$$\begin{array}{c} \begin{array}{c} \mu =\alpha \theta =\frac{1}{2}Nk_{B}T\\ \sigma =\sqrt{\alpha }\theta =\frac{1}{\sqrt{2}}\sqrt{N}k_{B}T\;. \end{array} \end{array}$$ (11)

Having means and averages consistent with the applied temperature is a necessary condition for a trajectory sampled from the correct distribution, which can easily be verified by calculating the empirical mean $\hat{\mu }$ and standard deviation $\hat{\sigma }$. A standard error estimate for these empirical quantities can be obtained by bootstrapping [33] the original trajectory. Estimates further than 2-3 standard errors away from the expected value in Eq 11 can then easily be flagged as violating physical assumptions. Furthermore, deviations in the mean or the standard deviation can intuitively be understood as “the average temperature is wrong” or “the distribution is too narrow / too wide”.

A possible cause for the failure to reproduce the expected mean and variance is lack of equipartition. Different sets of degrees of freedom may sample different distributions. As described earlier, this can easily be verified by comparing the empirical estimators for subsets of the system.

When assuming equipartition, the empirical moments can be directly related to temperatures. To see this, we plug the empirical estimates $\hat{\mu }$ and $\hat{\sigma }$ into Eq 11. As the principle of equipartition requires all degrees of freedom to be identically distributed, we can assume *N* to be constant. The equations then define two different empirical temperature for each moment,

$$\begin{array}{c} \begin{array}{c} T_{\hat{\mu }}=\frac{2\hat{\mu }}{Nk_{B}}\\ T_{\hat{\sigma }}=\frac{\sqrt{2}\hat{\sigma }}{\sqrt{N}k_{B}}\;. \end{array} \end{array}$$ (12)

These values can be understood as “the distribution has a mean equivalent to a physical distribution at $T_{\hat{\mu }}$” and “the distribution has a width equivalent to a physical distribution at $T_{\hat{\sigma }}$”. Note that this interpretations serve only the intuitive understanding. If the two values differ significantly, the statistical conclusion is therefore that the trajectory was not sampled from the physically expected distribution.

### Ensemble validation

The ratio of distributions of certain system observables (such as the energy, volume, or number of particles) for a system simulated at different state points define necessary conditions for the distribution of these observables in the system. As the distribution of configurational quantities like the potential energy *U*, the volume *V* (for the isothermal-isobaric (NPT) ensemble) or the number of each species ***N*** (for the grand-canonical (*μ*VT) ensemble) are in general not known analytically, testing the likelihood of a trajectory sampling a given ensemble is less straightforward than for the kinetic energy. However, the *ratio* of the probability distribution between samplings of the same system at different state points (e.g. at different temperatures, different pressures, different chemical potentials) can still often be useful to diagnose simulation errors. The logarithm of the probability densities of the configurational observables mentioned above is linearly dependent on the parameters [34],

$$\begin{array}{cc} \log\frac{P(E\;|\;\beta_{2})}{P(E\;|\;\beta_{1})}∼-(\beta_{2}-\beta_{1})E & \;\text{NVT} \end{array}$$ (13)

$$\begin{array}{cc} \log\frac{P(E,V\;|\;\beta_{2},P_{2})}{P(E,V\;|\;\beta_{1},P_{1})}∼-\left(\beta_{2}-\beta_{1}\right)E-\left(\beta_{2}P_{2}-\beta_{1}P_{1}\right)V & \;\text{NPT} \end{array}$$ (14)

$$\begin{array}{cc} \log\frac{P(E,N\;|\;\beta_{2},\mu_{2})}{P(E,N\;|\;\beta_{1},\mu_{2})}∼-\left(\beta_{2}-\beta_{1}\right)E+\sum_{i}\left(\beta_{2}\mu_{2,i}-\beta_{1}\mu_{1,i}\right)N_{i} & \;\mu \text{VT}\;. \end{array}$$ (15)

where *P*(*a* | *b*) denotes the probability of the observables *a* (the total energy *E*, or the volume *V* or species composition ***N***) in the ensemble defined by parameters *b*. The ensemble of the physical system is defined by the temperature *T*, and possibly the pressure *P* or the chemical potentials *μ*_*i*_. More details on the derivation are provided in S1 Text or Ref. [34].

Eqs 13–15 define an easily verifiable condition for trajectories. Given two (otherwise identical) simulations performed at different state points, our analysis tool therefore performs a maximum-likelihood analysis as described in Ref. [34] finding the most likely slope parameter(s) to fit Eqs 13–15. Under NVT conditions, only one parameter can be estimated, the difference in target temperature *β*_2_ − *β*_1_ between the two simulations. Under NPT conditions, three different cases with different parameters can occur:

1. If the two simulations differ only in target temperature, *β*_2_ − *β*_1_ is estimated by fitting to the distributions of the enthalpy *H* = *E* + *PV*;
2. if the two simulations differ only in target pressure, *P*_1_ − *P*_2_ is estimated by fitting to the distributions of the volume *V*; and
3. if the two simulations differ in both the target temperature and pressure, a two-dimensional fit estimating *β*_2_ − *β*_1_ and *β*_2_*P*_2_ − *β*_1_*P*_1_ simultaneously is performed.

Under *μ*VT conditions, three cases in analogy to the NPT case can be distinguished, namely

1. differing temperatures,
2. differing chemical potentials, and
3. a two-dimensional fit with differing temperatures and chemical potentials.

The assessment of the simulation quality can directly be tied to the estimated error of the fit. The standard deviation *σ* of the max-likelihood fit can be estimated in two ways: Analytically, by taking the square root of the diagonal of the Hessian of the log-likelihood function at the calculated minimum, or by bootstrap resampling. The quality of the simulation can then be assessed by calculating the number of standard deviations the estimate deviates from the expected value. The higher this number, the less likely it is that the observed deviation is solely due to noise. As a rule of thumb, a difference of more than about 3*σ* can be seen as a clear sign that some systematic error is causing the deviation.

Under NPT conditions, tests are currently only implemented for isotropic pressure conditions. An unambiguous definition of the probability distribution, and hence the partition function, of the ensemble is required to implement the test. For anisotropical pressure coupling, such a definition requires the proper definition of the work of the box deformation and treatment of the additional degrees of freedom due to the uncoupled box vectors. General literature descriptions of anisotropic barostats are often incomplete, and the exact distribution of box degrees of freedom appears to be dependent on the implementation details of the barostat. Implementing proper variants of nonisotropic barostats is therefore a future research question that will remain of interest to this effort.

The maximum-likelihood slope analysis also allows to estimate a more intuitive parameter interval. Under NVT or NPT conditions with two different temperatures *T*_1_ and *T*_2_, the slope *a*_*E*_ ≈ *β*_2_ − *β*_1_ approximates *β*_2_ − *β*_1_ = (*k*_*B*_*T*_2_)^−1^ − (*k*_*B*_*T*_1_)^−1^ = (*T*_1_ − *T*_2_)/(*k*_*B*_*T*_1_*T*_2_). We can hence define the interval estimate Δ*T*′ ≈ *T*_1_ − *T*_2_ as

$$\begin{array}{c} \Delta T^{\prime }=a_{E}k_{B}T_{1}T_{2}\;. \end{array}$$ (16)

Under NPT conditions with varying pressures but identical temperature *T*, the slope *a*_*V*_ ≈ *βP*_2_ − *βP*_1_ is already estimating the interval *P*_2_ − *P*_1_ up to the factor *β* = (*k*_*B*_*T*)^−1^,

$$\begin{array}{c} \Delta P^{\prime }=a_{V}k_{B}T\;. \end{array}$$ (17)

Under NPT conditions with varying pressures and temperatures, a two-dimensional fit is performed, with *a*_*E*_ estimating *β*_2_ − *β*_1_ and *a*_*V*_ estimating *β*_2_*P*_2_ − *β*_1_*P*_1_. Δ*T*′ is then calculated as defined in Eq 16, while the estimated pressure interval is approximated as

$$\begin{array}{c} \Delta P^{\prime }=a_{V}k_{B}\frac{T_{1}+T_{2}}{2}\;. \end{array}$$ (18)

In all cases, an error estimate for these intervals can easily be obtained via uncertainty calculation from the error estimate of the slope calculation. As the estimates in Eqs 16–18 are more intuitive than the pure slopes, they are reported along with the slopes, and used in the result section of this work.

It is often more useful to use only potential energy *U* instead of the total energy *E* in these calculations. As the kinetic energy *K* is independent of the configurational quantities in systems that properly obey statistical mechanics, Eqs 13–15 are fully valid also when replacing *E* by *U*. As described above, the kinetic energy can be checked (more rigorously) separately. For the same reason that the components of the kinetic energy may have incorrect distributions even though the total kinetic energy is correct, testing the total energy can mask compensating errors occurring in in the kinetic and potential energy.

#### Choice of temperature and pressure interval

As the ensemble tests presented above require two simulations at distinct state points, the choice of the interval between the two points becomes an important question. Choosing two state points too far apart will result in very small or zero overlap between the distributions, leading to very noisy results (due to sample errors in the tails) or a breakdown of the method, respectively. Choosing two state points very close to each other, on the other hand, makes it difficult to distinguish the slope from statistical error in the samples.

A rule of thumb states [34] that the maximal efficiency of the method is reached when the distance between the peaks of the distributions are roughly equal to the sum of their standard deviations. For most systems (with the notable exception of extremely small or very cold systems), it is reasonable to assume that the difference in standard deviations of the distributions will be negligible between state points located close to each others. This assumption leads to the following equations [34] for the suggested approximate state point intervals:

$$\begin{array}{c} \Delta T=\frac{2k_{B}T^{2}}{\sigma_{E}}\;, \end{array}$$ (19)

where *σ*_*E*_ is the standard deviation of the energy distribution used in the test (potential energy, enthalpy, or total energy), and

$$\begin{array}{c} \Delta P=\frac{2k_{B}T}{\sigma_{V}}\;, \end{array}$$ (20)

where *σ*_*V*_ is the standard deviation of the volume distribution.

The standard deviations themselves can be obtained in two ways. Given a simulation at one state point, and the previously mentioned assumption that the standard deviations will not significantly vary between the state points, a good interval for the second one can be estimated by simply calculating *σ*_*E*_ or *σ*_*V*_ (or both) from the simulated trajectories. Alternatively, the width of the distributions can be estimated from experimental observables or simulation observables. For the energy, the standard deviation can be estimated from the heat capacities,

$$\begin{array}{c} \begin{array}{c} \sigma_{E}^{2}=2k_{B}T^{2}C_{V}\;\text{(NVT)}\\ \sigma_{E}^{2}=2k_{B}T^{2}C_{P}\;\text{(NPT)}\;, \end{array} \end{array}$$ (21)

where *C*_*V*_ and *C*_*P*_ denote the isochoric and the isobaric heat capacities, respectively. For the volume, the standard deviation can be estimated from the isothermal compressibility *κ*_*T*_,

$$\begin{array}{c} \sigma_{V}^{2}=2k_{B}TV\kappa_{T}\;. \end{array}$$ (22)

In many cases, when using atomistic models, the experimental values will be sufficiently close to the simulated materials values that they can be used, since the range of intervals over which the test is statistically useful is relatively broad.

## Usage of the validation suite as an analysis tool

### Uniform representation

As different molecular mechanics (MM) packages store system information and simulation results in very different ways, a uniform representation is necessary to make analysis tools universally usable. While a general abstraction of MM data would be highly desirable, it is far beyond the scope of this work. The physical validation suite therefore defines a relatively simple Python class named SimulationData abstracting the data used by the different tests. Most tests presented need relatively simple information, such as the units and the time step used to ensure proper calculation, information on the temperature and pressure control and the number of degrees of freedom to select the right tests, and trajectories of energies, volume and pressure to perform the validation. The notable exception is the equipartition calculation, which in general needs full position and velocity trajectories to calculate kinetic energies of subsamples of the system, as well as information on the connectivity to distinguish single molecules.

To make the use of the validation suite as easy as possible, we have developed offering parsers generating SimulationData objects directly from the output file of simulation runs for a number of widely-used MM packages. Currently, parsers are available for GROMACS, LAMMPS, HOOMD, and GROMOS. For these packages, the creation of SimulationData representations reduces to a few Python instructions at most.

For simulation packages not supported to date, we offer the possibility to create SimulationData objects either from simple ASCII files, or from Python data structures. In both cases, information about units, the sampled ensemble and the system need to be provided by filling respective data structures by hand, as they cannot be read from simulation files directly. The observable, position and velocity trajectories can then be added by either reading in one- and three-dimensional “flat files” extracted from result files of MM programs, or by supplying Python data structures such as lists or numpy arrays obtained from the Python API of a simulation code or from other Python-based analysis tools.

Section A in S4 Text lists code snippets containing example usages of the different parsers.

### Result validation

Given one or multiple uniform SimulationData objects, running the validation tests presented in this work requires just a few function calls. Section B in S4 Text lists examples for validation of ensemble and integrator validation. The level of detail of the output of all tests can be tuned with the verbosity argument. Additionally, most tests can be visualized using the screen and the filename arguments, printing plots to screen and to file, respectively. Most tests also allow tuning the sensitivity by choosing a tolerance. The official documentation [35] contains references for all options and additional examples.

## Usage of the validation suite as a code validation tool

The tests presented in this work lay the groundwork for frameworks to test simulation codes for physical validity. Testing is an integral part of software development best practices. As physical and mathematical assumptions are fundamental to molecular simulation software, testing for their validity is a natural acceptance test.

Starting with version 2018, every major GROMACS releases is required to pass a set of physical validation tests covering important code paths. It is, to our knowledge, the first major molecular mechanics software package to run such validation routinely. The new physical validation tests thereby complement the established unit and regression tests.

The new tests aim at covering the most common code path and are built up in a hierarchical way. First, the available integrators are validated using the integrator tests on a system of mono-atomic Lennard-Jones (LJ) particles. These tests are then repeated on a system of TIP3P water molecules to involve electrostatic interactions and different bond treatments: constrained via LINCS or SETTLE, or using harmonic potentials. Further, the velocity-rescale and the Nosé-Hoover temperature control, and the Parrinello-Rahman and the MTTK pressure control are validated on the same systems using the presented ensemble tests for the kinetic and the configurational quantities. The tests are performed both in single and in double precision.

## Illustrative examples

### Discontinuities in potential and forces

**Fig 2:**
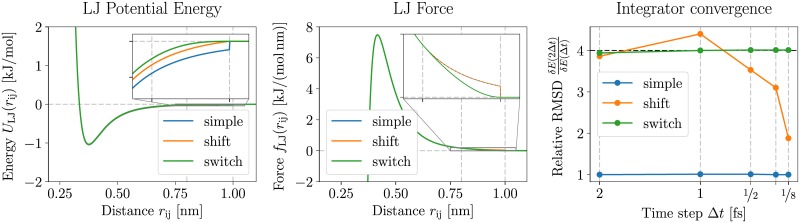
Integrator convergence test. The integrator convergence test picks up discontinuities in the forces or the potential energy. The data is reported numerically in Table A in S3 Text. Comparison of potential (left panel), force (central panel) and results from the integrator convergence test (right panel) for three different choices of cutoff schemes, with cutoff at 1 nm. The potential shows a discontinuity at the cutoff distance for the “simple” scheme (blue line), while the “shift” scheme (orange line) and the “switch” scheme (green line) are continuous. The forces, on the other hand, are only continuous in the “switch” scheme, while the other two schemes are discontinuous at the cutoff distance. The results of testing these three schemes with the integrator convergence validation presented earlier are depicted in the right panel, along with a horizontal dashed black line indicating the expected behavior. The “simple” scheme has no noticeable dependence of the fluctuations on the time step. The “shift” scheme is closer to the expected behavior at high timesteps, but loses the expected dependence for smaller timesteps. The “switch” scheme, finally, exhibits the expected convergence of the RMSD for all tested timestep sizes.

Using a Lennard-Jones (LJ) system, we demonstrate the ability of the integrator convergence test to detect discontinuities in the potential or the forces of a MD simulation. Such imprecisions in the interaction potential can arise from code errors or the choice of inappropriate parameters. To mimic these imprecisions, the LJ particles were simulated with three choices of interaction cutoff handling. Scheme “simple” cuts off the interaction at a defined cutoff radius, leading to discontinuities in both the potential and the forces. Scheme “shift” shifts the potential by a constant in order to ensure that the potential reaches zero at the interaction cutoff boundary, but leaves the forces unchanged. Scheme “switch” gradually switches the forces (and hence the potential) to zero in a buffer region close to the cutoff radius, ensuring both smooth forces and potential. The left and central panels in Fig 2 illustrate the potential and the forces for all schemes.

The simulations were performed using GROMACS 2016.4 compiled in double precision. The integrator test is naturally also valid in single precision. Notably, the GROMACS validation suite described earlier tests both the single and the double precision versions. The system consisted of 1000 LJ particles using Argon parameters by White [36] (*σ* = 0.3345 nm, *ϵ* = 1.045 128 kJ mol^−1^) in a cubic periodic box of length 3.6039 nm corresponding to a reduced density ρ* = 0.8. The LJ interactions were cut off at 1 nm, using either no modification of the interactions (scheme “simple”), using a potential shift (scheme “shift”) or switching the forces between 0.8 nm and 1 nm (scheme “switch”). The pairlist was calculated using the Verlet buffer scheme [37], with a tolerance of 10^−10^ kJ mol^−1^. We chose this unusually low tolerance in order to avoid any influence of the pairlisting algorithm on the simulation results. The initial box was created by randomly placing the particles in the computational box and randomly assigning velocities from a Maxwell-Boltzmann distribution at 125.707 K (reduced temperature T* = 1.0). The system was then minimized and equilibrated for 1 ns under NVE conditions. The final configuration of this equilibration run was then used to start the production simulations.

The production simulations consisted of 4 ps runs performed using six different timestep sizes (4 fs, 2 fs, 1 fs, ½ fs, ¼ fs, ⅛ fs). For each simulation, the total energy was saved every 4 fs. The root-mean-squared deviations (RMSD) of the total energy around its average value was then calculated and compared to the value obtained at half the timestep size. As detailed in earlier, the RMSD of a constant of motion is expected to be directly proportional to the square of the timestep. Consequently, when comparing a simulation to another (otherwise identical) simulation performed at half the timestep size, we expect the RMSD to decrease by a factor of 4.

We observed clear differences between the different cutoff schemes, and only the “switch” schemes passing the integrator test. The results are depicted in the right panel of Fig 2 and listed in Table A in S3 Text. The “simple” scheme does not display any dependence of the fluctuations on the timestep size, while the “shift” scheme starts deviating from the expected convergence at a timestep of ¼ fs. The error due to the cutoff treatment is of the order of magnitude of the RMSD at which the convergence starts failing. The “switch” scheme, on the other hand, shows little deviations from the expected convergence down to the lowest employed timestep of ⅛ fs.

The energy drift does not have a significant effect on these results. For the presented results, the fluctuations around the average energy measured over 4 ps were in all cases larger than the total drift over the 4 ps simulation by at least a factor of 10^3^ The influence of the long-term drift on the fluctuations in the short simulations can hence be assumed to be negligible. All drifts are listed in Table B in S3 Text.

### Validating temperature and pressure control algorithms

The combined ensemble validation tests for the kinetic energy and the configurational quantities allow validation of temperature and pressure control algorithms. We illustrate this on a simple water system simulated both under isochoric and isobaric boundary conditions. In both cases, the temperature was controlled using weak-coupling [38] (WC) or velocity-rescale [39] (VR) algorithm. For the isobaric simulations, these temperature-control algorithms were complemented by a weak-coupling or a Parrinello-Rahman [40] (PR) pressure control algorithm, respectively.

The simulations were performed using GROMACS 2018 compiled in double precision. The system consisted of 900 TIP3P water [41] molecules in a cubic box. The water molecules were kept rigid using the SETTLE [42] algorithm. The isochoric simulations were performed at a density of 986 kg m^−3^ and temperatures of 300 K and 308 K. The isobaric simulations were performed at four temperature / pressure points, 300 K / 1 bar, 308 K / 1 bar, 300 K / 301 bar, and 308 K / 301 bar. The pairwise interactions were cut off at 1 nm, with PME at 0.12 nm mesh size handling long-range interactions for both electrostatic and LJ interactions. The pairlist was calculated using the Verlet buffer scheme with a tolerance of 10^−10^ kJ mol^−1^. The initial box was created by randomly placing the water molecules in the computational box, minimizing the configuration, and randomly assigning velocities from a Maxwell-Boltzmann distribution at 300 K. The system was then equilibrated for 1 ns under NVE conditions, before running the integration validation test to exclude errors from other sources influencing the ensemble sampling. The simulations at the different state points were then equilibrated for 5 ns, before sampling for 15 ns using a timestep of 1fs and saving the energies every 1 ps. The sampled energies were then tested for equilibration and statistically decorrelated before further analysis. The coupling times for the temperature and pressure control algorithms were 0.1 ps (WC temperature), 1 ps (WC pressure), 0.1 ps (VR), and 2 ps (PR).

**Fig 3:**
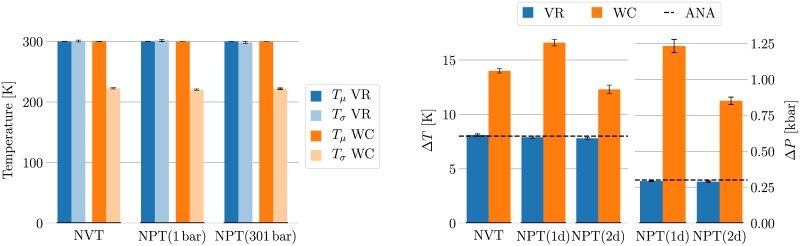
Kinetic energy and ensemble test. The coupling algorithms sample significantly different energy distributions. The data is reported numerically in Tables C and D in S3 Text. All results are from a water system of 900 TIP3P molecules. The temperature and pressure were controlled either using the VR algorithm in combination with a PR barostat (blue bars), or using the WC algorithm (orange bars). The left plot depicts the results of the non-strict kinetic energy test for simulations at 300 K. The distributions sampled by both algorithms are found to have the correct average temperature. The VR distribution also has the correct width, while the WC algorithms samples a distribution which is significantly too narrow in all cases. The right plot shows the intervals estimated by the ensemble check. The estimated temperature intervals for the VR algorithm lie all within 1.5 standard errors of the analytical value of 8 K (indicated by a dashed line). The WC algorithm, on the other hand, is estimated to have a temperature difference of 12.3 to 16.6 K, 11.4 to 34.6 standard errors from the true value. The pressure difference shows similar results. The true interval is 300 bar for the 1d-estimate, and 296.1 bar for the 2d-estimate. The VR estimates lie within 2.5 and 1.7 standard errors from the true value, respectively, while the WC estimates are found to be more than 20 standard errors from the true value.

We found that the different coupling schemes sample different kinetic energy ensembles. For all state points simulated, the *p*-value obtained from the K-S test under the null hypothesis that the kinetic energy is distributed according to Eq 10 is listed in Table 1. The trajectories obtained using the WC algorithm have a *p*-value smaller than 10^−82^ in all cases, making it statistically extremely unlikely that the expected ensemble was sampled. This is in agreement with the theoretical expectation that the WC algorithm does not sample a canonical distribution [13]. The results obtained with the VR/PR algorithms, on the other hand, have *p*-values above 0.23 in all cases, suggesting one should accept the null hypothesis that the kinetic energies are sampled from the expected distribution. The left panel of Fig 3 (numerical values in Table C in S3 Text) shows the temperature equivalent of the mean and variance as defined in Eq 12 for the simulations at 300 K. While both the WC and the VR algorithms keep the mean temperature close to 300 K, only the VR algorithm samples the expected width. The variance sampled with the WC thermostat is equal to the expected width around 220 K, and hence significantly too narrow.

**Table 1:** Strict kinetic energy test. Only the VR algorithm samples the expected kinetic energy distribution. This table lists the *p*-values of the kinetic energy validation. All results are from a water system of 900 TIP3P molecules. The temperature was controlled using either the weak-coupling (WC) or the velocity-rescale (VR) algorithm, complemented in the NPT case by a weak-coupling and a Parrinello-Rahman (PR) pressure-control algorithm, respectively. For all state points, the null hypothesis that the simulations controlled by the WC algorithms sampled the expected kinetic energy distribution can easily be rejected. For the simulations controlled by the VR/PR algorithms, however, the *p*-values indicate a high similarity between the sampled and the expected distribution.

	NVT		NPT			
	300 K	308 K	300 K	308 K	300 K	308 K
			1 bar	1 bar	301 bar	301 bar
**WC**	1.55 × 10^−88^	1.08 × 10^−90^	1.49 × 10^−93^	1.91 × 10^−89^	8.41 × 10^−83^	2.73 × 10^−86^
**VR**	0.29	0.88	0.90	0.86	0.23	0.30

Only using the VR/PR algorithms produced configurational quantities (potential energy, volume, enthalpy) that sampled the desired ensemble. Using Eqs 16–18, the difference in target temperature and / or pressure was calculated from the maximum-likelihood slope estimates. The uncertainty of this estimate was obtained by bootstrapping the original trajectory 200 times. Fig 3 (numerical values in Table D in S3 Text) shows the results of this ensemble checking for the different coupling schemes. The simulations controlled by the WC algorithms show a temperature- and pressure-dependency which varies significantly from the expected dependency. While the true temperature difference between the simulations was 8 K, the estimates based on the WC simulations range from 12.3 K to 16.6 K, 20.3 to 34.6 standard errors from the true value. The pressure-dependence does not yield better results, with estimates being up to 46 standard deviations away from the true value. On the other hand, the true temperature interval can be found within the standard deviation of the estimates obtained from the VR/PR simulations in all cases. The pressure estimates were found to be around 2 standard deviations from the true value, indicating roughly satisfactory agreement with the expected ensemble.

### Validating equipartition

Validating the equipartition allows detection of unsuitable simulation setups significantly affecting physical observables. This is illustrated by calculating the heat of vaporization Δ*H*_vap_ of a set of eight linear alcohols with increasing chain length (methanol to octanol) using the 2016H66 parameter set [43]. See the first 8 rows of Table 1 in Ref. [43] for details on the test set.

Calculating Δ*H*_vap_ requires two simulations of the model, one in condensed phase and one in gas phase.Δ*H*_vap_ is calculated as the difference between the average energy in the gas and the liquid phase increased by *RT*,

$$\begin{array}{c} \Delta H_{\text{vap}}={\left\langle U\right\rangle }_{\text{gas}}-{\left\langle U\right\rangle }_{\text{liq}}+RT\;. \end{array}$$ (23)

Different simulation setups to calculate the gas phase estimate were previously reported in the literature. Here, we compare three methods: *(i)* Using stochastic dynamics (SD) implementation of the Langevin equations of motion [44, 45], as in Ref. [43], *(ii)* using MD and a WC thermostat as in Refs. [46, 47], and *(iii)* using MD and a VR thermostat.

Most settings were identical for all simulations, adapting the GROMOS protocol in Ref. [43] to fit the GROMACS 2018 standards. The pairlist was calculated using the Verlet buffer scheme with a tolerance of 5 × 10^−3^ kJ mol^−1^ and a pairwise interaction cut-off radius of 1 nm. For the liquid simulations, PME at 0.12 nm mesh size was used to handle long-range effects for both electrostatic and LJ interactions. For the gas-phase simulations, a straight cut-off with potential-shift was used for both electrostatic and LJ interactions. All simulations used a timestep of 2 fs.

All simulations were initialized by randomly placing 512 molecules in cubic boxes of appropriate size for the experimental density (see Ref. [43], Table 1) and performing energy minimization on the obtained system. The following equilibration and liquid phase production runs were performed under NPT conditions at 298.15 K and 1 bar. The temperature was controlled using the v-rescale algorithm, while the pressure was controlled with a Parrinello-Rahman barostat. The minimized configurations were equilibrated for 1 ns. The liquid phase were then simulated for 2 ns, saving the energies every 2 ps for further analysis.

Starting from the equilibrated configuration, the gas phase was simulated by turning off all inter-molecular interactions, mimicking an ideal gas. The pressure control was turned off, keeping the volume constant. The temperature was, as mentioned above, controlled by either controlling the temperature in MD using the VR or the WC thermostat, or by integrating the SD equations of motion.

**Fig 4:**
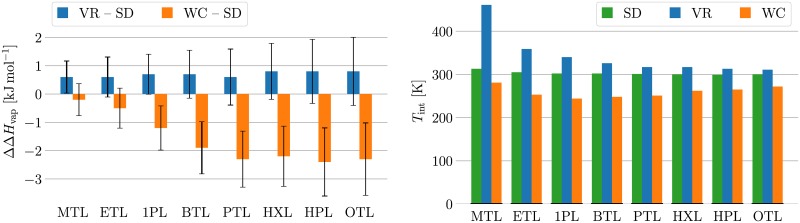
Equipartition of Δ*H*_vap_ estimates. Systematic differences between Δ*H*_vap_ estimates from different gas-phase setups for a range of linear alcohols from methanol to octanol are due to violation of equipartition. The data is reported numerically in Table E in S3 Text. The compound abbreviations are listed in Table E in S3 Text and in Ref. [43]. The left plot depicts the differences between two MD protocols using the VR and the WC thermostats, respectively, to the SD protocol. Compared to the SD setup, the WC setup is found to yield estimates that are consistently too low, while the VR setup yields estimates that are too high. The deviation between the WC setup and the SD setup lies outside the sum of the standard errors for the compounds with alkyl chains of three or more carbon atoms. The deviation between the VR and SD setup lies within the sum of the standard errors. The right plot depicts the internal temperatures averaged over the simulation runs for all three gas-phase setups, as well as for the liquid setup. The total temperature is kept at 298K in all cases. The internal temperatures of the SD setup are very close to the target value and virtually indistinguishable from the liquid simulations. The VR results show a significantly increased internal temperature, while the WC results show significantly too cold internal temperatures.

The gas-phase setup significantly influences the estimate of the heat of vaporization. As can be seen in the left panel of Fig 4, the three schemes yield different Δ*H*_vap_ estimates. Compared to SD, the WC estimates are systematically lower, and the difference between the estimates is around three standard errors for the longer alcohols with four or more carbon atoms in the alkyl chain. VR, on the other hand, yields estimates which are systematically higher than the SD estimates. The difference between the two estimates is around one standard error in all cases. Please refer to Table E(a) in S3 Text for the numerical Δ*H*_vap_ estimates.

The difference in Δ*H*_vap_ estimates is due to violation of equipartition in the MD schemes. Fig 4 plots the average internal temperature of the three gas-phase schemes. Please refer to Table E(b) in S3 Text for the full numerical details on the total, translational, rotational and internal temperatures averaged over the simulation runs for all three gas-phase schemes and the liquid simulation. Unsurprisingly, the average total temperature is 298 K in all cases. The liquid simulations show a slightly reduced rotational temperature, which is compensated by a slightly elevated internal temperature. The SD gas-phase simulations follow this trend. The WC gas-phase simulations display increased translational (in all cases but methanol) and rotational temperatures, and reduced temperatures for the internal degrees of freedom. The VR scheme, on the other hand, exhibits reduced translational and rotational (in all cases but methanol), but increased internal temperatures. This analysis shows that the SD scheme is the only gas-phase scheme preserving equipartition to a similar extent as the liquid simulations, and should hence be preferred for the calculation of gas-phase estimates. (Note that while the gas-phase estimates in Refs. [46, 47] were produced using an MD scheme potentially violating equipartition, correspondence with the respective authors revealed that they drew new velocities from a Maxwell distribution every 50ps. This led to estimates that show no significant deviation from SD results, and hence did not negatively influence the quality of the published force field parameters).

The choice of kinetic energy estimator does not significantly influence these conclusions, as the deviations due to the decoupling of the degrees of freedom in the gas phase simulations is larger than the influence of the finite timestep on the temperature estimate. To document this, we have have recalculated the equipartition for one molecule using robust temperature estimators proposed by Eastwood *et al*. [27]. The results are listed in Table F in S3 Text and show that the difference in equipartition due to the choice of temperature estimator is negligible compared to the deviations due to the choice of thermostat.

### Validating coupling schemes

Equipartition tests make it possible to determine the appropriate coupling scheme for a given calculation. When determining how to couple a system to an external bath, the main criterion must be a correct physical description. As an example, we apply different coupling schemes to a Trp-cage mini-protein solvated in water. We used three different temperature-control algorithms, the VR and the WC algorithms used previously, and the Nose-Hoover thermostat [13, 48] (denoted NH), all using a coupling time *τ*_*T*_ = 0.1 ps. All thermostats were applied in three different ways: coupling the entire system to a single heat bath (subscript _1_), coupling the solute and the solvent separately (subscript _2_), and coupling only the solvent to a bath (subscript _*s*_), yielding 9 different coupling schemes.

The simulations were performed with GROMACS 2016.3. The Trp-cage parameters were taken from the OPLS [49, 50] forcefield. The Trp-cage configuration was minimized and solvated in 5228 TIP3P water molecules. One chloride ion was added to neutralize the total charge of the solute. The pairlist was generated using the Verlet algorithm with a tolerance of 0.005 kJ mol^−1^. The pairwise interactions were cut off at 1 nm, using PME to approximate their long-range effects. The equations of motion were integrated using the leap-frog algorithm with a timestep of 2 fs. All bonds were constrained using the LINCS [51, 52] algorithm. The pressure was kept around 1 bar using the Parrinello-Rahman barostat. The thermostats were used to keep the temperature around 300 K. For all schemes, the minimized configuration was equilibrated for 1 ns, before a production run of 10 ns was started, saving the energies, the atom coordinates and the atom velocities every 2 ps for further analysis.

**Fig 5:**
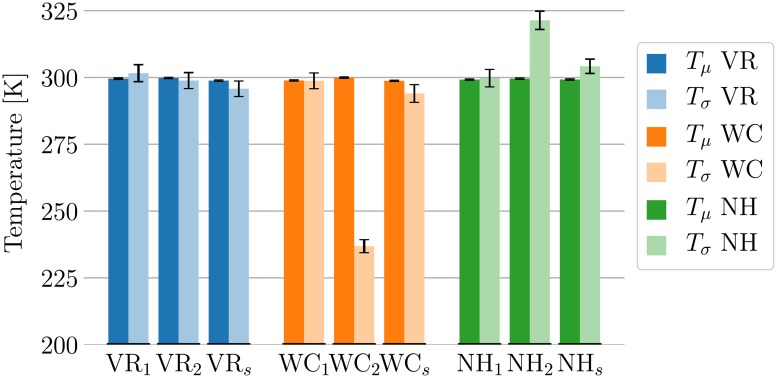
Validation of temperature coupling schemes for Trp-cage. The width of the kinetic energy distributions sampled by the different schemes differ significantly when the solute is coupled to a separate thermostat. The data is reported numerically in Table G in S3 Text. This plot lists the temperature equivalent (see Eq 12) of the mean and the variance of the internal temperature of the Trp-cage peptide for nine different thermostat coupling schemes. All coupling schemes keep the average temperature close to its target value of 300K. When using a single thermostat to couple the entire system, the width of the distribution is very close to the expected value for all thermostats. When coupling the solute to a separate thermostat, the VR algorithm samples the expected distribution. The WC algorithm is found to sample a distribution which is clearly too narrow, while the NH thermostat samples a slightly too wide distribution. When coupling only the solvent to a heat bath, small deviations of low statistical significance can be observed in all cases.

Examining the temperature of the solute degrees of freedom, we find clear differences between the coupling schemes. The system used here has about 500 times more solvent atoms than solute atoms. The kinetic energy of the system will hence be dominated by the solvent. For typical applications, however, the distribution of the solute is far more important than the one of the solvent. This energy distribution should therefore be checked to evaluate validity of different system setups. In typical MD codes, the center of mass is usually artificially removed, as with periodic boundary conditions, this degree of freedom is nonphysical. Although in principle this should remain zero in a constant energy simulation due to conservation of momentum, any source of numerical error, including roundoff error, will create motion around the center of mass, and any variation of Langevin dynamics will also introduce center of mass motion. This center of mass removal will therefore also remove three degrees of freedom from the system. When analyzing only a part of the system, there is however no guarantee that the center of mass of the subsystem is exactly zero. This includes the case presented here, in which we are looking at a single solute molecule. Additionally, as described in earlier, the kinetic energy distribution of the internal degrees of freedom is required to ensuring correct system properties. We will therefore focus on the internal kinetic energy for the subsystems for the remainder of this discussion. Fig 5 shows the mean and the width of the distribution of the internal temperature of the solute. The values are given in terms of temperatures *T*(*μ*) and *T*(*σ*), as described in Eq 12. The numerical values are listed in Table G in S3 Text, along with the total temperature of the solute. As can be verified there, the internal and the total temperature do not differ significantly in this case.

When using a single thermostat coupling the entire system, the protein shows the correct kinetic energy distribution in all cases. It is especially noteworthy that the WC_1_ algorithm, albeit sampling the wrong kinetic energy distribution on a full system, shows the correct behavior properties when looking at the solute only. This is in agreement with previous findings [53].

Using two thermostats, the different algorithms all keep the mean temperature close to the target value, but show significant differences in the width of the sampled distribution. The NH_2_ thermostat samples a slightly too wide distribution, equal to the width expected at a temperature of about 321 K. On the other hand, the WC_2_ algorithm shows a too narrow distribution as observed earlier, having a width as expected for a temperature of about 237 K. The distribution generated by the VR_2_ scheme shows the correct width.

Coupling only the solute, slight deviations in the width of the sampled distributions can be observed. All schemes keep the average temperature of the solute close to the target value through energy exchange. The width of the distribution sampled by the NH_*s*_ scheme is slightly too high (corresponding to about 304 K), while the WC_*s*_ and VR_*s*_ schemes sample slightly to small variances (corresponding to about 294 K and 296 K, respectively. These deviations have, however, low statistical significance, as they are below 2 standard errors from the true value in all cases.

## Conclusions

In this paper, we have reviewed a number of important physical laws that valid molecular simulations must satisfy, and demonstrated how these physical laws can be checked using a number of simple tests, as well as presented an easy-to-use physical validation software suite to assess molecular simulation correctness and improve simulation reproducibility. The increasing versatility of simulation programs and the availability of APIs makes it very difficult for developers to test all possible combinations of parameters and simulation conditions. Additionally, even a bug-free program does not guarantee that the physical validity is maintained under all conditions. The wide availability of high-performance computing resources attracts many users with very different backgrounds to the field of molecular simulations. It is paramount that reliable simulation results can be obtained without years of experience in molecular simulation. The tests and software suite we present implementing these tests can help solve both problems.

We have shown how checking the timestep dependence of energy conservation can detect even subtle inaccuracies in the simulation protocol. A deviation from the mathematically expected quadratic dependence of the energy fluctuations on the timestep is a sensitive test for the health of a simulation setup. It is hence a powerful test to ensure that correctness is maintained when altering the program code. We have shown how this approach can detecting subtle differences in consistency with Newton’s equations of motion caused by different cutoff schemes for Lennard-Jones interactions.

We have also shown how testing the distribution of the kinetic energy allows to ensure that the expected ensemble is sampled. The kinetic energy distribution is analytically known, and can hence be checked using standard statistical methods. This results in a very sensitive test of the validity of temperature control algorithms and the associated simulation setup. We used this test to reproduce the well-known result that weak-coupling thermostat does not yield the expected statistical mechanical distribution, while the addition of a stochastic term in the v-rescale algorithm allows to remedy this short-coming. For many real-world applications, it is sufficient and more intuitive to ensure that the kinetic energy has the proper average value and distribution width. We have presented a less strict test relating these values to temperatures. Using this test, we have compared the results of simulations of a mini-protein in water using different temperature coupling schemes. We showed that the kinetic energy distribution of the solute is nearly indistinguishable when using different thermostats, including the weak-coupling algorithm, when coupling the entire system to a single thermostat.

Not only the kinetic energy of the entire system but the kinetic energy of the components of the systems must satisfy a well-prescribed distribution. We have demonstrated how to divide the kinetic energy in translational, rotational and internal components. We have then used this division to explain why gas-phase estimates obtained using different temperature-controlling algorithms deviate significantly from each others.

While the distribution of other ensemble quantities are not known analytically in general, the difference of their distributions at different state points can be used to check the sampled ensemble. Using these tests, we have demonstrated that the thermostats not only sample different kinetic energy distributions, but also significantly alter the distribution of the potential energy and the volume.

We have developed a Python package that implements the tests for testing a simulation run’s physical validity. This package is open-source and platform-independent. It supports a number of simulation packages natively (GROMACS, LAMMPS, HOOMD, GROMOS), and allows to use results obtained from other sources either via text files or Python data structures. We will continue to maintain this package in the near future, adding more tests and support for additional simulation packages.

Lastly, we have implemented these physical validity checks in the software testing routines of GROMACS. Since GROMACS 2018, every major release is required to pass a set of physical validation tests, adding additional coverage to the already existing unit and regression tests.

## Supporting information

S1 Text Derivation of ensemble ratios. (PDF)

S2 Text Separation of molecular degrees of freedom. (PDF)

S3 Text Additional results. (PDF)

S4 Text Script examples. (PDF)
