# Supplementary material for: Testing for physical validity in molecular simulations
Source: PLoS One. 2018 Sep 6;13(9):e0202764. doi: 10.1371/journal.pone.0202764 (PMC6126824; doi:10.1371/journal.pone.0202764)
Supplement: S1 Text — (PDF) [file pone.0202764.s001.pdf]

S1 Derivation of ensemble ratios

Following Ref. [1] the analytical ratio between observable distributions at two different state points can be used to verify the sampled ensemble. Specifically, the phase-space probability densities of the canonical (NVT), the isothermal-isobaric (NPT), and the grand-canonical (μVT) have similar mathematical forms,

$$P(\boldsymbol{r}, \boldsymbol{p} \mid \beta) \sim e^{-\beta \mathcal{H}(\boldsymbol{r}, \boldsymbol{p})} \qquad \text{NVT} \tag{1}$$

$$P(\boldsymbol{r}, \boldsymbol{p}, V \mid \beta, P) \sim e^{-\beta \mathcal{H}(\boldsymbol{r}, \boldsymbol{p}) - PV} \qquad \text{NPT} \tag{2}$$

$$P(\boldsymbol{r}, \boldsymbol{p}, \boldsymbol{N} \mid \beta, \boldsymbol{\mu}) \sim e^{-\beta \mathcal{H}(\boldsymbol{r}, \boldsymbol{p}) + \sum_i \beta \mu_i N_i} \qquad \mu\text{VT}\,, \tag{3}$$

where $P(a \mid b)$ denotes the probability of microstate a in the ensemble defined by parameters b. The microstate is defined by the positions $\boldsymbol{r}$ and momenta $\boldsymbol{p}$ of the atoms in the system, and potentially the volume V (for NPT) and the number of particles N_i for each species i (for μVT). The ensemble of the physical system is defined by the temperature T, the pressure P, the chemical potentials μ_i and the functional form of the Hamiltonian $\mathcal{H}(\boldsymbol{r}, \boldsymbol{p})$.

The probability of observing a specific total energy $E = \mathcal{H}(\boldsymbol{r}, \boldsymbol{p})$ (in NVT), a specific total energy E along with a specific volume V (in NPT) or a specific total energy E along with a specific species composition $\boldsymbol{N}$ (in μVT) is then given by

$$P(E \mid \beta) \sim \Omega(E)\; e^{-\beta E} \qquad \text{NVT} \tag{4}$$

$$P(E, V \mid \beta, P) \sim \Omega(E, V)\; e^{-\beta E - PV} \qquad \text{NPT} \tag{5}$$

$$P(E, \boldsymbol{N} \mid \beta, \boldsymbol{\mu}) \sim \Omega(E, \boldsymbol{N})\; e^{-\beta E + \sum_i \beta \mu_i N_i} \qquad \mu\text{VT}\,, \tag{6}$$

where Ω denotes the density of states, which is generally a non-trivial, system-dependent quantity. The presence of Ω explains why no direct statistical tests for the probability distribution of configurational quantities exist.

Ω is, however, independent from the state parameters of the ensemble, as can be verified by looking at at Eqs. 4 – 6. The ratios of the probability densities of observables of the identical system at different state points are hence independent of Ω,

$$\frac{P(E \mid \beta_2)}{P(E \mid \beta_1)} \sim e^{-(\beta_2 - \beta_1) E} \qquad \text{NVT} \tag{7}$$

$$\frac{P(E, V \mid \beta_2, P_2)}{P(E, V \mid \beta_1, P_1)} \sim e^{-(\beta_2 - \beta_1) E - (\beta_2 P_2 - \beta_1 P_1) V} \qquad \text{NPT} \tag{8}$$

$$\frac{P(E, \boldsymbol{N} \mid \beta_2, \boldsymbol{\mu}_2)}{P(E, \boldsymbol{N} \mid \beta_1, \boldsymbol{\mu}_2)} \sim e^{-(\beta_2 - \beta_1) E + \sum_i (\beta_2 \mu_{2,i} - \beta_1 \mu_{1,i}) N_i} \qquad \mu\text{VT}\,. \tag{9}$$

Taking the logarithm of these ratios, it becomes obvious that in all cases, a linear dependence of the observables exist:

$$\log \frac{P(E \mid \beta_2)}{P(E \mid \beta_1)} \sim -(\beta_2 - \beta_1) E \qquad \text{NVT} \tag{10}$$

$$\log \frac{P(E, V \mid \beta_2, P_2)}{P(E, V \mid \beta_1, P_1)} \sim -(\beta_2 - \beta_1) E - (\beta_2 P_2 - \beta_1 P_1) V \qquad \text{NPT} \tag{11}$$

$$\log \frac{P(E, \boldsymbol{N} \mid \beta_2, \boldsymbol{\mu}_2)}{P(E, \boldsymbol{N} \mid \beta_1, \boldsymbol{\mu}_2)} \sim -(\beta_2 - \beta_1) E + \sum_i (\beta_2 \mu_{2,i} - \beta_1 \mu_{1,i}) N_i \qquad \mu\text{VT}\,. \tag{12}$$

References

1. Shirts MR. Simple Quantitative Tests to Validate Sampling from Thermodynamic Ensembles. Journal of Chemical Theory and Computation. 2013;9(2):909–926. doi:10.1021/ct300688p.
