# Supplementary material for: Testing for physical validity in molecular simulations
Source: PLoS One. 2018 Sep 6;13(9):e0202764. doi: 10.1371/journal.pone.0202764 (PMC6126824; doi:10.1371/journal.pone.0202764)
Supplement: S2 Text — (PDF) [file pone.0202764.s002.pdf]

S2 Separation of molecular degrees of freedom

The total kinetic energy of a molecular system of particles is given by

$$K_{\mathrm{tot}} = \frac{1}{2} \sum_i m_i \boldsymbol{v}_i^2 \,, \tag{1}$$

where the sum runs over all atoms i in the system, mass m_i and $\boldsymbol{v}_i$ being the mass and velocity vector of atom i, respectively. The component of the kinetic energy associated with the center-of-mass motion translation of the molecules is calculated as

$$K_{\mathrm{tra}} = \frac{1}{2} \sum_J M_J \boldsymbol{v}_J^2 \,, \quad \text{with } \boldsymbol{v}_J = \frac{1}{M_J} \sum_{i \in J} m_i \boldsymbol{v}_i \,, \tag{2}$$

where the sum runs over all molecules J in the system, M_J and $\boldsymbol{v}_J$ being the total mass and translational center-of-mass velocity vector, respectively. The rotational and internal kinetic energy is then simply the remainder,

$$K_{\mathrm{r+i}} = K_{\mathrm{tot}} - K_{\mathrm{tra}} \,, \tag{3}$$

or

$$K_{\mathrm{r+i}} = \frac{1}{2} \sum_i m_i \boldsymbol{v}_{\mathrm{r+i},i}^2 \,, \quad \text{with } \boldsymbol{v}_{\mathrm{r+i},i} = \boldsymbol{v}_i - \boldsymbol{v}_{J(i)} \,. \tag{4}$$

This kinetic energy can be further separated into rotational and internal components [1]. The rotational part represents the rotational kinetic energy of a hypothetical rigid body having the shape of the molecule in the current configuration, whereas the internal part contains any deviation from the rigid-body behavior. The instantaneous tensor of inertia of molecule J is defined by its components as

$$\begin{aligned} I_{J,\alpha\alpha} &= \sum_{i \in J} m_i (\boldsymbol{r}_{\mathrm{r+i},i}^2 - \alpha_{\mathrm{r+i},i}^2) \\ I_{J,\alpha\beta} &= -\sum_{i \in J} m_i \alpha_{\mathrm{r+i},i} \beta_{\mathrm{r+i},i} \,, \end{aligned} \tag{5}$$

where $\alpha, \beta \in \{x, y, z\}$ with $\alpha \neq \beta$, the components of $\boldsymbol{r}$ are $\{x, y, z\}$, and $\boldsymbol{r}_{\mathrm{r+i},i}$ is the position of atom i relative to the center of mass of the molecule J it belongs to. The angular momentum $\boldsymbol{L}_J$ vector of molecule J is given by

$$\boldsymbol{L}_J = \sum_{i \in J} m_i \boldsymbol{r}_{\mathrm{r+i},i} \times \boldsymbol{v}_{\mathrm{r+i},i} \,, \tag{6}$$

and is related to the angular velocity vector $\boldsymbol{\omega}_J$

$$\boldsymbol{L}_J = \underline{\boldsymbol{I}}_J \boldsymbol{\omega}_J \,. \tag{7}$$

The rotational kinetic energy is then given by

$$K_{\mathrm{rot}} = \frac{1}{2} \sum_J \boldsymbol{\omega}_J^T \boldsymbol{L}_J = \frac{1}{2} \sum_J \boldsymbol{\omega}_J^T \underline{\boldsymbol{I}}_J \boldsymbol{\omega}_J \,. \tag{8}$$

The internal kinetic energy is then simply given by the difference between the rotational and internal kinetic energy (Eq. 4) and its purely rotational component (Eq. 8), namely

$$K_{\mathrm{int}} = K_{\mathrm{r+i}} - K_{\mathrm{rot}} \,. \tag{9}$$

References

1. Jellinek J, Li DH. Separation of the Energy of Overall Rotation in Any N-Body System. Physical Review Letters. 1989;62(3):241–244. doi:10.1103/PhysRevLett.62.241.
