# Supplementary material for: Testing for physical validity in molecular simulations
Source: PLoS One. 2018 Sep 6;13(9):e0202764. doi: 10.1371/journal.pone.0202764 (PMC6126824; doi:10.1371/journal.pone.0202764)
Supplement: S3 Text — (PDF) [file pone.0202764.s003.pdf]

S3 Additional results

	simple		shift		switch	
Δt [fs]	$\delta E(\Delta t)$ [kJ mol^{-1}]	$\frac{\delta E(2\Delta t)}{\delta E(\Delta t)}$	$\delta E(\Delta t)$ [kJ mol^{-1}]	$\frac{\delta E(2\Delta t)}{\delta E(\Delta t)}$	$\delta E(\Delta t)$ [kJ mol^{-1}]	$\frac{\delta E(2\Delta t)}{\delta E(\Delta t)}$
4	7.08×10^{-1}	–	1.03×10^{-2}	–	9.90×10^{-3}	–
2	7.12×10^{-1}	1.00	2.68×10^{-3}	3.86	2.51×10^{-3}	3.94
1	7.07×10^{-1}	1.01	6.09×10^{-4}	4.40	6.28×10^{-4}	4.00
$1/2$	7.02×10^{-1}	1.01	1.73×10^{-4}	3.53	1.57×10^{-4}	4.01
$1/4$	7.02×10^{-1}	1.00	5.56×10^{-5}	3.10	3.91×10^{-5}	4.01
$1/8$	7.04×10^{-1}	1.00	2.96×10^{-5}	1.88	9.76×10^{-6}	4.01

Table A. Results of the integrator convergence test. Only the system using a cutoff with switched forces passes the integrator test. The data is illustrated graphically in Fig. 2. This table lists numerical results of the integrator convergence test for three different choices of cutoff schemes. For each of the three schemes “simple”, “shifted” and “switch” at all tested timestep sizes, the table lists fluctuations of the total energy (δE), and the ratio of the fluctuations at the next-larger timestep and the current timestep ($\frac{\delta E(2\Delta t)}{\delta E(\Delta t)}$). It can be observed that for the “simple” scheme, the fluctuation ratio is found to be around 1 in all cases, indicating no dependence of the fluctuations on the chosen timestep. For the “shift” scheme, a dependence on the timestep can be observed, which is around the expected value of 4 for the higher timesteps, but decreases as the lowest tested timesteps are reached. The “switch” scheme, on the other hand, exhibits the expected dependence on the timestep even at the smallest timestep tested.

	Slope of total energy [kJ mol^{-1} / ns]		
Δt [fs]	**simple**	**shift**	**switch**
4	8.56×10^{-4}	1.16×10^{-4}	9.64×10^{-6}
2	-1.55×10^{-3}	4.95×10^{-5}	-2.85×10^{-7}
1	2.29×10^{-3}	9.73×10^{-5}	-8.23×10^{-7}
$^1/_2$	-5.06×10^{-4}	-1.85×10^{-5}	-1.27×10^{-7}
$^1/_4$	1.73×10^{-3}	8.11×10^{-6}	-3.90×10^{-7}
$^1/_8$	-2.13×10^{-4}	-7.92×10^{-8}	-4.54×10^{-8}

Table B. Energy drift of the constant energy simulations. This table lists the energy drift of the constant energy simulations for the three different cutoff schemes “simple”, “shifted” and “switch” at all tested timestep sizes. The drift is calculated by performing a least-squares fit of the total energy trajectory over 1 ns to a straight line. The drift is in all cases significantly smaller than the RMSD reported in Tab. A.

	NVT			
	300 K		308 K	
	T_μ	T_σ	T_μ	T_σ
**WC**	300.00 ± 0.03	222.66 ± 1.10	307.99 ± 0.03	229.10 ± 1.19
**VR/PR**	299.95 ± 0.04	300.71 ± 1.52	308.05 ± 0.05	309.12 ± 1.77

	NPT							
	300 K, 1 bar		308 K, 1 bar		300 K, 301 bar		308 K, 301 bar	
	T_μ	T_σ	T_μ	T_σ	T_μ	T_σ	T_μ	T_σ
**WC**	299.95 ± 0.03	220.45 ± 1.12	307.97 ± 0.03	227.82 ± 1.24	299.93 ± 0.03	222.01 ± 1.18	307.94 ± 0.03	226.48 ± 1.29
**VR**	300.04 ± 0.05	301.64 ± 1.75	307.99 ± 0.05	306.59 ± 1.64	300.01 ± 0.05	298.22 ± 1.58	307.97 ± 0.05	309.96 ± 1.78

Table C. Results of the non-strict kinetic energy test. The coupling algorithms sample significantly different kinetic energy distributions. The data is illustrated graphically in Fig. 3. All results are from a water system of 900 TIP3P molecules. The temperature was controlled using either the weak-coupling (WC) or the velocity-rescale (VR) algorithm, complemented in the NPT case by a weak-coupling and a Parrinello-Rahman (PR) pressure-control algorithm, respectively. This table lists the empirical temperatures for the mean and the variance, as defined by Eq. 12. It becomes obvious that the mean of both the WC and the VR distributions is correct, but that WC samples a distribution which is significantly narrower than expected.

	NVT		NPT *(1d)*				NPT *(2d)*			
	ΔT [K]	$\#\sigma$	ΔT [K]	$\#\sigma$	ΔP [bar]	$\#\sigma$	ΔT [K]	$\#\sigma$	ΔP [bar]	$\#\sigma$
**ANA**	8.0		8.0		300.0		8.0		296.1	
**WC**	14.0 ± 0.2	34.6	16.6 ± 0.3	33.4	1232.7 ± 46.0	20.3	12.3 ± 0.4	11.4	851.5 ± 25.3	22.0
**VR/PR**	8.1 ± 0.1	0.7	7.9 ± 0.1	0.7	292.7 ± 3.5	2.1	7.8 ± 0.1	1.5	288.7 ± 4.4	1.7

Table D. Results of the ensemble test. The coupling algorithms sample significantly different potential energy distributions. The data is illustrated graphically in Fig. 3. All results are from a water system of 900 TIP3P molecules. The temperature was controlled using either the weak-coupling (WC) or the velocity-rescale (VR) algorithm, complemented in the NPT case by a weak-coupling and a Parrinello-Rahman (PR) pressure-control algorithm, respectively. This table lists interval estimates at different state points as described in the theory section of the main text. The row ANA lists the expected (analytical) intervals. The rows WC and VR/PR list the interval estimates obtained by comparing the simulations at different temperatures under NVT and NPT (ΔT, one-dimensional temperature fit), the simulations at different pressures under NPT (ΔP, one-dimensional pressure fit), and simulations differing in both temperature and pressure under NPT (ΔT and ΔP simultaneously, two-dimensional fit). Each simulation estimate is followed by the number of standard deviations ($\#\sigma$) the estimate deviates from the true value.

(a) Heat of vaporization estimates

	ΔH_{vap} [kJ mol^{-1}]		
Comp.	WC	VR	SD
MTL	42.5 ± 0.4	43.3 ± 0.4	42.7 ± 0.4
ETL	45.6 ± 0.5	46.7 ± 0.5	46.1 ± 0.5
1PL	49.6 ± 0.6	51.5 ± 0.5	50.8 ± 0.5
BTL	54.0 ± 0.7	56.6 ± 0.6	55.9 ± 0.6
PTL	58.8 ± 0.7	61.7 ± 0.7	61.1 ± 0.7
HXL	64.1 ± 0.8	67.1 ± 0.7	66.3 ± 0.7
HPL	69.1 ± 0.9	72.3 ± 0.8	71.5 ± 0.8
OTL	74.3 ± 1.0	77.4 ± 0.9	76.6 ± 0.8

(b) Average of total, translational, rotational and internal temperature (all values in [K])

	GAS WC				GAS VR				GAS SD				LIQ VR			
Comp.	T_{tot}	T_{tra}	T_{rot}	T_{int}	T_{tot}	T_{tra}	T_{rot}	T_{int}	T_{tot}	T_{tra}	T_{rot}	T_{int}	T_{tot}	T_{tra}	T_{rot}	T_{int}
MTL	298	269	336	273	298	240	303	459	298	298	294	309	298	297	294	313
ETL	298	321	327	246	298	250	285	357	299	299	296	301	298	297	294	303
1PL	298	375	340	226	298	248	270	343	298	299	291	302	298	297	291	303
BTL	298	375	350	243	298	257	278	324	298	299	296	299	298	297	295	300
PTL	298	391	360	247	298	268	277	316	298	298	296	299	298	298	296	299
HXL	298	377	366	258	298	251	280	316	298	298	296	299	298	298	297	299
HPL	298	377	379	261	298	250	284	312	298	300	296	298	298	298	297	299
OTL	298	385	361	269	298	260	277	310	298	298	296	298	298	297	296	299

Table E. Equipartition of ΔH_{vap} estimates. Systematic differences between ΔH_{vap} estimates from different gas-phase setups are due to violation of equipartition. The data is illustrated graphically in Fig. 4. The test set includes eight linear alcohols — methanol (MTL), ethanol (ETL), propanol (1PL), butanol (BTL), pentanol (PTL), hexanol (HXL), heptanol (HPL), and octoanol (OTL). (a) lists ΔH_{vap} estimates for all compounds, computed using three different gas-phase simulation setups. Compared to the SD setup, the WC setup is found to yield consistently lower estimates, while the VR setup yields higher estimates. The deviation between the WC setup and the SD setup is around three standard errors for the compounds with alkyl chains of four or more carbon atoms. The deviation between the VR and SD setup is around one standard error in all cases. (b) lists the total, translational, rotational and internal temperatures averaged over the simulation runs for all three gas-phase setups, as well as for the liquid setup. The total temperature is kept at 298 K in all cases. The liquid simulations show a slightly elevated internal temperature and a slightly reduced rotational temperature. The gas-phase results of the SD setup are virtually indistinguishable from the liquid simulations. The VR results show a significantly increased internal temperature, while the WC results show significantly too cold internal temperatures.

Estimator	GAS WC T_{tot}	GAS WC T_{tra}	GAS WC T_{rot}	GAS WC T_{int}	GAS VR T_{tot}	GAS VR T_{tra}	GAS VR T_{rot}	GAS VR T_{int}
T_{p^2}	298.03	374.65	339.80	225.91	297.93	248.37	269.52	343.02
$T_{pv}^{(2)}$	296.80	374.66	340.09	223.82	296.42	248.93	269.31	339.19
$T_{pv}^{(4)}$	297.54	374.66	340.11	225.43	297.48	247.93	270.18	341.71
$T_{pv}^{(6)}$	297.70	374.66	340.16	225.48	297.72	247.95	270.11	341.60

Table F. Equipartition using different temperature estimates. The difference between equipartition estimates using different temperature estimates is negligible compared to the deviations due to the choice of thermostat. This table lists the equiparition for propanol (1PL) estimated by different temperature estimates: Calculated from the integrated momenta only (T_{p^2}), or from the product of the integrated momenta and an estimate of the time derivative of second order ($T_{pv}^{(2)}$), fourth order ($T_{pv}^{(4)}$), or sixth order ($T_{pv}^{(6)}$), as defined by Eastwood *et al.* [1]. The more robust temperature estimates $T_{pv}^{(2)}$, $T_{pv}^{(4)}$, and $T_{pv}^{(6)}$ do display only slightly different temperatures compared to the standard estimate T_{p^2}. Most notably, the differences between the more robust estimates and the standard estimates are much smaller than the deviations from the expected equipartition behavior.

Scheme	$T_{\mathrm{p,tot}}(\mu)$ [K]	$T_{\mathrm{p,tot}}(\sigma)$ [K]	$T_{\mathrm{p,int}}(\mu)$ [K]	$T_{\mathrm{p,int}}(\sigma)$ [K]
VR_1	299.53 ± 0.23	301.46 ± 3.30	299.57 ± 0.23	301.62 ± 3.18
VR_2	299.76 ± 0.26	298.85 ± 3.13	299.81 ± 0.27	298.83 ± 3.00
VR_s	298.81 ± 0.24	295.77 ± 2.70	298.82 ± 0.25	295.78 ± 2.91
WC_1	298.85 ± 0.25	298.83 ± 2.98	298.88 ± 0.25	298.74 ± 2.95
WC_2	299.93 ± 0.18	236.79 ± 2.43	299.95 ± 0.19	236.85 ± 2.45
WC_s	298.69 ± 0.24	294.59 ± 2.90	298.74 ± 0.24	294.00 ± 3.31
NH_1	299.12 ± 0.23	299.54 ± 2.91	299.20 ± 0.24	299.74 ± 3.27
NH_2	299.52 ± 0.26	321.26 ± 3.57	299.56 ± 0.27	321.41 ± 3.43
NH_s	299.19 ± 0.24	303.60 ± 2.93	299.23 ± 0.26	304.19 ± 2.67

Table G. Validation of temperature coupling schemes for trp-cage. The width of the kinetic energy distributions sampled by the different schemes differ significantly when the solute is coupled to a separate thermostat. The data is illustrated graphically in Fig. 5. This table lists the temperature equivalent (see Eq. 12) of the mean and the variance of the total and the internal temperature of the trp-cage peptide for nine different thermostat coupling schemes.

References

1. Eastwood MP, Stafford KA, Lippert RA, Jensen MØ, Maragakis P, Predescu C, Dror RO, Shaw, DE. Equipartition and the Calculation of Temperature in Biomolecular Simulations. Journal of Chemical Theory and Computation. 2010;7:2045–2058. doi:10.1021/ct9002916.
