# Supplementary material for: Testing for physical validity in molecular simulations
Source: PLoS One. 2018 Sep 6;13(9):e0202764. doi: 10.1371/journal.pone.0202764 (PMC6126824; doi:10.1371/journal.pone.0202764)
Supplement: S4 Text — (PDF) [file pone.0202764.s004.pdf]

S4 Script examples

A Create simulation result representation

A.1 GROMACS parser

For GROMACS, a typical code snippet creating a `SimulationData` object looks as follows:

```
import physical_validation as pv

gmx_parser = pv.data.GromacsParser(
    exe='~/bin/gromacs/bin/gmx',
    includepath='~/bin/gromacs/share/gromacs/top'
)
res = gmx_parser.get_simulation_data(
     mdp='mdout.mdp',
     top='system.top',
     gro='system.gro',
     edr='system.edr'
)
```

Listing 1. Creating simulation data using the `GromacsParser`

After the import statement, the first command in Lst. 1 initializes a parser object with a GROMACS executable and, optionally, the path to the topology include library. The executable is used to call GROMACS programs such as `gmx energy`, while the topology path is used to resolve `#include` statements in topologies. The second command creates the simulation data representation from GROMACS input and output files.

A.2 Creation from flat files or Python data structures

For simulation packages not supported to date, information about units, the sampled ensemble and the system need to be provided by filling respective data structures by hand, as they cannot be read from simulation files directly. Lst. 2 first creates data structures for a system with 900 rigid water molecules using GROMACS units. Given these data structures, one possibility is to create `SimulationData` objects from "flat files", simple one- or three-dimensional ASCII files containing observable or position / velocity trajectories from simulations. The `FlatfileParser` offers an easy way to include simulation results from custom codes into analysis scripts in that way. The other possibility to create `SimulationData` objects is directly from Python data structures. This allows to use lists or numpy arrays obtained from the Python API of a simulation code, or from other Python-based analysis tools.

```
import physical_validation as pv

system = pv.data.SystemData(
    natoms=900*3,
    nconstraints=900*3,
    ndof_reduction_tra=3,
    ndof_reduction_rot=0
)
units = pv.data.UnitData.units('GROMACS')
ensemble = pv.data.EnsembleData(
    ensemble='NVT',
    natoms=900*3,
    volume=3.01125**3,
    temperature=298.15
)

# kinetic.dat, potential.dat and total.dat are files
# containing the energies of a simulation run
flat_parser = pv.data.FlatfileParser()
res_flat = flat_parser.get_simulation_data(
    units=units, ensemble=ensemble, system=system,
    kinetic_ene_file='kinetic.dat',
    potential_ene_file='potential.dat',
    total_ene_file='total.dat'
)

# kin_ene, pot_ene and tot_ene are lists or numpy
```

```
# arrays containing the energies of a simulation run
observables = pv.data.ObservableData(
    kinetic_energy = kin_ene,
    potential_energy = pot_ene,
    total_energy = tot_ene
)
res_py = pv.data.SimulationData(
    units=units, ensemble=ensemble,
    system=system, observables=observables
)
```

Listing 2. Creating simulation data objects from flat files or Python data structures

B Validate simulation results

B.1 Validate ensembles

```
import physical_validation as pv

# Create simulation data representation at different state points
gmx_parser = pv.data.GromacsParser(
    exe='~/bin/gromacs/bin/gmx',
    includepath='~/bin/gromacs/share/gromacs/top'
)
res_low = gmx_parser.get_simulation_data(
     mdp='low/mdout.mdp',
     top='low/system.top',
     gro='low/system.gro',
     edr='low/system.edr'
)
res_high = gmx_parser.get_simulation_data(
     mdp='high/mdout.mdp',
     top='high/system.top',
     gro='high/system.gro',
     edr='high/system.edr'
)

# Validate kinetic energy distribution
pv.kinetic_energy.mb_ensemble(
    res_low, alpha=0.05, verbosity=1,
    filename='plot_low_mb'
)
pv.kinetic_energy.mb_ensemble(
    res_high, alpha=0.05, verbosity=1,
    filename='plot_high_mb'
)

# Validate configurational ensemble
pv.ensemble.check(
    res_low, res_high, verbosity=1,
    filename='plot_ensemble'
)
```

Listing 3. Validating ensemble of GROMACS simulation

B.2 Validate integrator convergence

```
import physical_validation as pv

# Create simulation data representation at different timesteps
gmx_parser = pv.data.GromacsParser(
    exe='~/bin/gromacs/bin/gmx',
    includepath='~/bin/gromacs/share/gromacs/top'
)
res_dt = []
for dir in ['dt_1', 'dt_2', 'dt_3', 'dt_4']:
    res_dt.append(
        gmx_parser.get_simulation_data(
            mdp='high/mdout.mdp',
```

```
            top='high/system.top',
            gro='high/system.gro',
            edr='high/system.edr'
        )
    )

# Validate convergence of integrator
pv.integrator.convergence(res, verbosity=1,
                          filename='plot_integrator')
```

Listing 4. Validating integrator of GROMACS simulation
